# Low probability of disease cure in advanced ovarian carcinomas before the PARP inhibitor era

**DOI:** 10.1038/s41416-022-01732-7

**Published:** 2022-03-31

**Authors:** Benoit You, Lilian Van Wagensveld, Michel Tod, Gabe S. Sonke, Hugo M. Horlings, R. F. P. M. Kruitwagen, Andreas Du Bois, Frédéric Selle, Timothy Perren, Jacobus Pfisterer, Florence Joly, Adrian Cook, Marie Christine Kaminsky, Kerstin Wollschlaeger, Alain Lortholary, Oliver Tome, Alexandra Leary, Gilles Freyer, Maaike Van Der Aa, Olivier Colomban

**Affiliations:** 1 Univ Lyon; Université Claude Bernard Lyon 1; Faculté de médecine Lyon-Sud; EA UCBL/HCL 3738 CICLY; Pharmacie, Lyon, France; 2 Medical Oncology; Institut de Cancérologie des Hospices Civils de Lyon (IC-HCL); CITOHL; Centre Hospitalier Lyon-Sud; GINECO, Lyon, France; 3 Department of Research and Development, Netherlands Comprehensive Cancer Organization (IKNL), Utrecht, the Netherlands; 4 Department of Obstetrics and Gynecology, Maastricht University Medical Centre, Maastricht, the Netherlands; GROW, School for Oncology and Developmental Biology, Maastricht, the Netherlands; 5 Hospices Civils de Lyon; Pharmacie; Hôpital de la Croix Rousse, Lyon, France; 6 Department of Medical Oncology, The Netherlands Cancer Institute, Amsterdam, the Netherlands; 7 Department of Pathology, The Netherlands Cancer Institute, Amsterdam, the Netherlands; 8 Kliniken Essen-Mitte (KEM); Essen; AGO Study Group, Essen, Germany; 9 Groupe Hospitalier Diaconesses Croix Saint-Simon, Department of medical oncology, and GINECO, Paris, France; 10 Leeds Institute of Medical Research at St James’s University Hospital, Leeds, UK; 11 Gynecologic Oncology Center, Herzog-Friedrich-Str. 21; 24103 Kiel; AGO Study Group, Kiel, Germany; 12 Centre François Baclesse; Medical Oncology Department; GINECO, Caen, France; 13 Medical Research Council Clinical Trials Unit at University College London, Aviation House, 125 Kingsway, London, UK; 14 Institut de Cancérologie de Lorraine; GINECO, Vandœuvre-lès-Nancy Cedex, France; 15 Otto-von-Guericke University Hospital; Department of Gynecology and Obstetrics; Magdeburg; AGO Study Group, Magdeburg, Germany; 16 Hôpital privé du confluent, GINECO, Nantes, France; 17 ViDia Christliche Kliniken Karlsruhe; Department of Gynecology and Obstetrics; Karlsruhe; AGO Study Group, Karlsruhe, Germany; 18 Institut Gustave Roussy, GINECO, Villejuif, France

**Keywords:** Medical research, Ovarian cancer

## Abstract

**Background:**

In ovarian carcinomas, the likelihood of disease cure following first-line medical-surgical treatment has been poorly addressed. The objective was to: (a) assess the likelihood of long-term disease-free (LDF) > 5 years; and (b) evaluate the impact of the tumour primary chemosensitivity (assessed with the modelled CA-125 KELIM) with respect to disease stage, and completeness of debulking surgery.

**Methods:**

Three Phase III trial datasets (AGO-OVAR 9; AGO-OVAR 7; ICON-7) were retrospectively investigated in an “adjuvant dataset”, whilst the Netherlands Cancer Registry was used in a “neoadjuvant dataset”. The prognostic values of KELIM, disease stage and surgery outcomes regarding the likelihood of LDF were assessed using univariate/multivariate analyses.

**Results:**

Of 2029 patients in the “adjuvant dataset”, 82 (4.0%) experienced LDF (Stage I–II: 25.9%; III: 2.1%; IV: 0.5%). Multivariate analyses identified disease stage and KELIM (OR = 4.24) as independent prognostic factors. Among the 1452 patients from the “neoadjuvant dataset”, 36 (2.4%) had LDF (Stage II–III: 3.3%; IV: 1.3%). Using multivariate tests, high-risk diseases (OR = 0.18) and KELIM (OR = 2.96) were significant.

**Conclusion:**

The probability of LDF > 5 years after first-line treatment in 3486 patients (<4%) was lower than thought. These data could represent a reference for future studies meant to assess progress related to PARP inhibitors.

## Introduction

The majority of patients (~75%) with high-grade carcinomas are diagnosed at advanced Stages III–IV [1]. The standard treatment in the first-line setting has historically relied on the combination of debulking surgery and systemic medical therapy. In addition, maintenance treatment with poly(ADP-ribose) polymerase inhibitors (PARPi) and/or bevacizumab as maintenance treatment was recently introduced [2–4].

The strong prognostic value of the completeness of debulking surgery has largely been reported and structured in the disease management guidelines. More recently, potential indicators of the tumour primary platinum sensitivity were described, including the ELIMination rate constant K (KELIM), based on the longitudinal kinetics of CA-125 during the first 100 days of first-line platinum-based chemotherapy [5]. KELIM, calculated with the mathematical equation driving the CA-125 longitudinal kinetics (≥3 values) during the first three to four cycles of neoadjuvant or adjuvant chemotherapy, has been developed to obtain an accurate characterisation of the CA-125 dynamics. The reliability of KELIM as an independent indicator of tumour platinum-based chemosensitivity has been reproducibly shown in many studies with more than 12,000 patients [6–12]. These studies have confirmed the capacity of KELIM to reproducibly predict: (1) the likelihood of complete resection at IDS in the neoadjuvant setting, (2) the probability of subsequent platinum-resistant relapse, (3) the patient PFS and OS.

The main purpose of medical-surgical treatment is to maximise the likelihood of obtaining a disease cure. It is considered that ~70% of patients with epithelial ovarian cancers will experience disease relapse, with numbers varying according to disease stages (from 10% at Stage I to 90% at Stage IV) (https://www.cancer.org/cancer/ovarian-cancer/detection-diagnosis-staging/survival-rates.html). However, the probability of cure has actually been poorly addressed.

The objective was to assess the likelihood of disease cure, explored with the rate of long-term disease-free (LDF) ≥ 5 years after first-line treatment, and evaluate the respective parts of (1) the tumour primary chemosensitivity, (2) disease stage and (3) the completeness of debulking surgery, relative to the success of the medical-surgical treatment, before the emergence of PARPi.

## Methods

Three large randomised Phase III datasets encompassing 2868 patients treated with the standard first-line carboplatin-paclitaxel (CP) regimen with/without a third agent (AGO-OVAR 9, CP ± gemcitabine; AGO-OVAR 7, CP ± topotecan; and ICON-7 trials, CP ± bevacizumab), previously analysed for KELIM investigation [6], were used to build an “adjuvant dataset” of 2029 assessable patients (70.7%) with Stage I–IV diseases, treated with primary debulking surgery and adjuvant chemotherapy. LDF was defined as the absence of disease progression or death within the first 5 years. Patients were assessable if they had experienced disease progression or death, or if they were free-of-progression or death with a minimum 5-year follow-up. The Netherlands Cancer Registry (NCR) composed of 1582 patients with Stage II–IV diseases treated with neoadjuvant chemotherapy potentially followed by interval debulking surgery (IDS) was used to build a “neoadjuvant dataset” with 1452 assessable patients (91.8%) [10].

The calculation of individual KELIM values in these datasets was previously reported [6, 10].

Descriptive statistics, along with univariate and multivariate logistic regressions were performed to assess the prognostic values of pathological subtypes; treatment arms; disease stage combined to the completeness of IDS in order to separate high-risk diseases (Stage IV, or incompletely resected Stage III diseases) and low-risk diseases in the NCR; and individual standardised (std) KELIM (considered as a continuous covariate; or categorised as a score: unfavourable if <1, or favourable if ≥1). BRCA mutational status was available for a small percentage of patients enrolled in the NCR. To explore the prognostic value of KELIM with respect to BRCA mutational status, univariate and multivariate analyses were performed in the subgroup of patients with known BRCA mutational status.

To account for the limited number of patients with LDF and potential biases related to the exclusion of patients without progression events within the first 5 years, censored quantile regressions were performed in order to assess the effects of these parameters on the distributions of progression-free survival (PFS) events [13].

All assessed studies (AGO-OVAR 7, AGO-OVAR 9, ICON-7 and NCR) were conducted in accordance with the Declaration of Helsinki ethical guidelines. All patients recruited in the study signed an informed written consent.

## Results

**Fig. 1:**
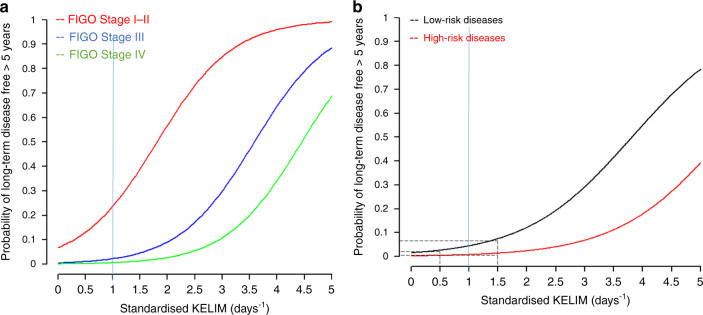
Probability of long-term disease free according to the tumor intrinsic chemosensitivity. Multivariate logistic regression model of the probability of long-term disease-free (LDF) > 5 years according to: **a** disease stage and standardised KELIM in the “adjuvant dataset”; **b** to disease-risk group (based the disease stage and the completeness of interval debulking surgery) and to standardised KELIM in the “neoadjuvant dataset” (Netherlands Cancer Registry). For example, for a patient with a Stage III disease, the probability of LDF is estimated at 7.0% in the case of complete IDS & favourable std KELIM at 1.5 days^−1^; 3.0% in the case of complete IDS & unfavourable std KELIM at 0.5 days^−1^; 2.0% in the case of incomplete IDS & favourable std KELIM at 1.5 days^−1^; and 1.0% in the case of incomplete IDS & favourable std KELIM at 0.5 days^−1^. Dashed black line: cut-off for unfavourable KELIM < 1; or favourable KELIM ≥ 1. *LCR* Long complete remission.

The characteristics of assessable patients are presented in Table 1. Out of 2029 patients in the “adjuvant dataset” (median PFS, 13.8 months, 95% CI 13.3–14.2; median overall survival, 37.9 months, 95% CI 36.8–39.8), 82 patients (4.0%) experienced LDF (45-month median follow-up). As expected, the probability of LDF decreased in higher disease stages, from 25.9% in Stage I–II, to 2.1% in Stage III, and 0.5% in Stage IV. The median std KELIM was significantly higher among patients who experienced LDF (1.14 versus 0.81 days^−1^, *P* < 0.01). Among 641 patients with favourable std KELIM (31.5%), 7.9% experienced LDF, ranging from 34.9% in Stage I–II to 4.5% in Stage IV. Using logistic regression models, two significant independent prognostic factors were associated with the likelihood of LDF: disease stage (Stage I–II, reference; Stage III, odds ratio (OR) = 0.07, 95% confidence interval (CI) 0.04–0.12; Stage IV, OR = 0.02, 95% CI 0.00–0.07); and std KELIM (OR = 4.24, 95% CI 2.36–7.69) (Fig. 1a). Censored quantile regression of PFS distribution showed that the highest evaluable decile (6th decile) of the failure-time of patients with favourable was higher by 23.0 months (95% CI 13.4–29.9) compared to those with unfavourable KELIM.

**Table 1:** Characteristics of included patients.

	Adjuvant dataset			Neoadjuvant dataset		
Number of patients assessed for KELIM in previous studies	2868			1582		
Number of patients assessable for long complete remission > 5 years	2029 (70.7%)			1452 (91.8%)		
Long complete remission > 5 years	Yes	No	Total	Yes	No	Total
	N (%)	N (%)	N	N (%)	N (%)	N
Pathological subtypes						
Serous	57 (3.6.%%)	1517 (96.4%)	1574	24 (2.2%)	1047 (97.8)	1071
Other subtypes	25 (5.5%)	427 (94.4%)	452	12 (3.2%)	369 (96.8%)	381
Disease stage						
Stage I–II	48 (25.9%)	137 (74.1%)	185	2 (Stage II only) (22.2%)	7 (Stage II only) (77.8%)	9 (Stage II only)
Stage III	32 (2.1%)	1448 (97.9%)	1480	26 (3.1%)	811 (96.9%)	837
Stage IV	2 (0.5%)	362 (99.5%)	364	8 (1.3%)	598 (98.7%)	606
Surgery outcomes						
Complete surgery with no residual lesions	NA	NA	NA	29 (5.7%)	476 (94.3%)	505
Incomplete surgery with residual lesions	NA	NA	NA	4 (0.6%)	602 (99.4%)	606
Standardised KELIM	Median 1.14 [1.02–1.20]	Median 0.81 [0.78–0.83]	///	Median 1.54 (1.34–1.79]	Median 1.04 [1.01–1.08]	///
Favourable ≥1.0 days^−1^	51 (7.9%)	590 (92.1%)	641	29 (3.6%)	767 (96.4%)	796
Unfavourable <1.0 days^−1^	31 (2.2%)	1357 (97.8%)	1388	7 (1.0%)	649 (99.0%)	656
Total	82 (4.0%)	1947 (96.0%)	2029	36 (2.4%)	1416 (97.6%)	1452
BRCA mutational status						
BRCA1 mutation	NA	NA	NA	2 (3.1%)	62 (96.9%)	64
BRCA2 mutation	NA	NA	NA	5 (16.7%)	25 (83.3%)	30
BRCA wild-type	NA	NA	NA	7 (1.9%)	348 (98.1%)	355
Missing	NA	NA	NA	22 (2.1%)	981 (97.9%)	1003

*NA* not available.

Among the 1452 patients in the “neoadjuvant dataset” (median PFS, 12.1 months, 95% CI 11.6–12.4; median overall survival, 23.1 months, 95% CI 21.8–24.2), 36 patients (2.4%) experienced LDF (95-month median follow-up) (Table 1). Similarly, to the “adjuvant dataset”, the percentages of patients experiencing LDF were higher among patients with favourable KELIM, regardless of disease stage (Table 1). Using logistic regression models, two independent prognostic factors were significantly associated with the likelihood of LDF: disease-risk group (high-risk versus low-risk, OR = 0.18, 95% CI 0.07–0.38); and std KELIM (OR = 2.96, 95% CI 1.46–5.90) (Fig. 1B). Censored quantile regressions of PFS distribution showed that the highest evaluable decile (8th decile) of the failure-time of patients with favourable was higher by 12.0 months compared to unfavourable KELIM (95% CI 8.2–17.7), and lower by −25.1 months (95% CI −39.7 to −17.6) for those with high-risk versus low-risk diseases.

The germline BRCA mutational status was available in 449 patients of the NCR (30.9%) (Table 1). BRCA1 mutation did not exhibit any prognostic value in univariate analysis (yes versus no, OR = 1.0, 95% CI 0.15–3.79). Among patients with a BRCA2 mutation (*n* = 30 patients), LDF were observed in a higher percentage of patients (16.7%), regardless of KELIM. In the multivariate analysis, standardised KELIM was not significantly associated with survival when tested together with high-risk disease (yes versus no, OR = 0.28, 95% CI 0.08–0.95) and BRCA2 mutation (yes versus no, OR = 1.78, 95% CI 1.46–21.03), suggesting that BRCA2 mutation integrates the information about the tumour’s intrinsic chemosensitivity.

## Discussion

The actual risk of disease relapse after first-line treatment is a subject of controversies. Indeed very few studies addressed this question, and inconsistent numbers were reported in the literature, ranging from 17 to 80% [14, 15].

This study composed of two large independent datasets, provides new data about the determinants of the first-line treatment success before the PARPi era. The present study showed that the probability of disease cure after first-line treatment was much lower than thought within the scientific community. In patients with Stage III and IV, representing ~75% of cases at diagnosis, the rates of LDF were only ~3%, and ~1%, respectively. As expected, the overall prognosis of patients treated with neoadjuvant chemotherapy and interval debulking surgery was worse than those treated with primary debulking surgery, in terms of PFS, and OS especially. Nevertheless, the probability of long-term disease-free was not very different between the two datasets, thereby meaning both endpoints are not necessarily related.

Interestingly, our data show that these probabilities are 3.5 times higher in patients with favourable KELIM compared to those with unfavourable KELIM, suggesting a major role of tumour primary chemosensitivity. The multivariate logistic regression models confirmed that the tumour primary chemosensitivity exhibited an independent prognostic value, together with the disease stage and the completeness of IDS.

These results should be analysed with caution due to significant limitations. Among them, the selection of the LDF as a potential indicator of disease cure is highly debatable. Approximately 22% of patients were not assessable regarding this endpoint (29% for the “adjuvant dataset” and 8% for the “neoadjuvant dataset”). To account for the potential biases related to the exclusion of these patients, the censored quantile regressions of all patient PFS distributions confirmed the impact of KELIM on the highest PFS deciles. Moreover, the present study is limited by the heterogeneity of patient characteristics and medical-surgical treatments, the low numbers of patients with Stage I–II diseases, along with the lack of data about the completeness of surgery and BRCA mutational status in the “adjuvant dataset”. The BRCA mutational status was available for only 31% of patients of IKNL registry (449 patients), meaning that the data about links between KELIM and BRCA1 or BRCA2 mutations are still very exploratory. Recent data on patients enrolled in the SOLO-1, PAOLA-1 and PRIMA trials suggest that the probability of LDF will improve with PARPi in the future [16]. It is likely that the patients with BRCA2 mutations will derive the highest benefit from these therapeutics [17–19]. A part of this effect may be related to the higher tumour primary chemosensitivity related to BRCA2 mutation, as suggested here with KELIM.

The present data could be used as a reference for future studies meant to assess the progress related to the introduction of PARPi in the first-line setting.

## Supplementary information

Checklist
